# Convergence of Cortical and Sensory Driver Inputs on Single Thalamocortical Cells

**DOI:** 10.1093/cercor/bht173

**Published:** 2013-07-03

**Authors:** Alexander Groh, Hajnalka Bokor, Rebecca A. Mease, Viktor M. Plattner, Balázs Hangya, Albrecht Stroh, Martin Deschenes, László Acsády

**Affiliations:** 1 Institute of Neuroscience, Technische Universität München, D-80802 Munich, Germany; 2 Laboratory of Thalamus Research,; 3 Laboratory of Cerebral Cortex Research, Institute of Experimental Medicine, Hungarian Academy of Sciences, Budapest H-1083, Hungary; 4 Focus Program Translational Neurosciences (ftn) & Institute for Microscopic Anatomy and Neurobiology, Johannes Gutenberg-University Mainz, D-55128 Mainz, Germany; 5 Centre de Recherche Université Laval Robert-Giffard, Laval University, Québec City, Canada G1J 2G3

**Keywords:** layer 5B, posterior-medial nucleus, relay, somatosensory, supralinear, top–down processing, trigeminal

## Abstract

Ascending and descending information is relayed through the thalamus via strong, “driver” pathways. According to our current knowledge, different driver pathways are organized in parallel streams and do not interact at the thalamic level. Using an electron microscopic approach combined with optogenetics and in vivo physiology, we examined whether driver inputs arising from different sources can interact at single thalamocortical cells in the rodent somatosensory thalamus (nucleus posterior, POm). Both the anatomical and the physiological data demonstrated that ascending driver inputs from the brainstem and descending driver inputs from cortical layer 5 pyramidal neurons converge and interact on single thalamocortical neurons in POm. Both individual pathways displayed driver properties, but they interacted synergistically in a time-dependent manner and when co-activated, supralinearly increased the output of thalamus. As a consequence, thalamocortical neurons reported the relative timing between sensory events and ongoing cortical activity. We conclude that thalamocortical neurons can receive 2 powerful inputs of different origin, rather than only a single one as previously suggested. This allows thalamocortical neurons to integrate raw sensory information with powerful cortical signals and transfer the integrated activity back to cortical networks.

## Introduction

Thalamic activity is indispensable for normal cortical function (Steriade et al. 1997; Jones 2007). Consequently, clarifying the principles of thalamic computation is necessary to understand cortical operations. According to our current view, the thalamus relays distinct sensory signals via segregated thalamic pathways to the cortex (Andersson et al. 1966; Friedman and Jones 1981; Jones et al. 1982, for a review see Steriade et al. 1997). For example, in the somatosensory system of rodents, signals evoked by whisker motion and touch are transferred via distinct thalamic cell populations (Yu et al. 2006; Diamond et al. 2008). Similarly, in the visual system inputs from retinal ganglion cells with different receptive field properties (X and Y cells) are relayed via separate thalamocortical cells (Friedlander et al. 1981; Hamos et al. 1987; Usrey et al. 1999), despite the fact that the 2 retinal inputs innervate the same region in the thalamus (Wilson et al. 1976).

In the present model of information flow through the thalamus, separation of parallel channels is achieved by a simple wiring principle, a single thalamic relay cell is innervated by a single type of sensory input (Steriade et al. 1997). These afferents form few, but powerful, giant terminals (drivers), which establish several synapses on the proximal dendrites of thalamic relay cells. As a result of these morphological properties, driver terminals are able to evoke fast-rising, large amplitude postsynaptic responses and postsynaptic spikes in the thalamic neurons which ensure faithful information transfer (Sherman and Guillery 1998). Drivers carrying distinct types of messages do not converge in the thalamus; these messages are rather integrated at the level of sensory cortices (Sherman and Guillery 2006).

Recently, it has become clear that driver pathways do not only originate in brainstem centers but can also arise from the neocortex. Axon collaterals of layer 5 (L5) pyramidal neurons establish giant excitatory terminals in “higher order” thalamic nuclei and display pharmacological properties and short-term plasticity similar to those observed in subcortical drivers (Rouiller and Welker 2000; Reichova and Sherman 2004; Groh et al. 2008). Thus, the thalamus is apparently innervated both by bottom-up and top-down drivers, hence the question arises whether subcortical and cortical drivers are kept separate or, alternatively, if these 2 signal streams interact in single neurons.

Previously, cortical and subcortical driver pathways have been shown to target the same thalamic nucleus in the somatosensory thalamus of rodents (Hoogland et al. 1991; Lavallee et al. 2005). According to the prevailing view about segregated information channels in the thalamus, even if these 2 distinct drivers innervate the same zone, they will not converge on individual thalamic neurons. However, this hypothesis has never been formally tested. In this study, we asked whether L5 input can converge with subcortical inputs on single thalamocortical neurons, which would allow the sensory cortex to exert a powerful control on the relay of ascending sensory messages.

## Materials and Methods

All experiments were done according to the guidelines of the German, Hungarian, and Canadian animal welfare and were approved by the respective ethical committees.

### Anatomical Experiments

Adult male Thy1 ChR (line 18) mice or adult male Wistar rats were injected iontophoretically through glass micropipettes (*d* = 50 μm) with phaseolus vulgaris leucoagglutinin (PHAL) (Vector Laboratories, 2.5% in 0.01 M phosphate buffer (PB), 5 μA, 7 s on/off duty cycle, 20 min; *n* = 5 mice and *n* = 6 rats) or biotinylated dextran amine (BDA) (2 μA, 2 s on/off, 20 min, pipette *d* = 15 μm, *n* = 4 rats). Coordinates for rats: Bregma −1.2 mm, lateral −5.0, and 1.5 mm ventral form cortical surface; mice: Bregma −1.2 mm, lateral −3.0, and 0.8 mm ventral form cortical surface. After a surviving period of 5–7 days, the mice were perfused with a fixative containing 4% paraformaldehyde (PF) and 0.1% glutaraldehyde in PB (0.1 M phosphate buffer). The rats were perfused with a 2-component fixative; first with 2% PF 0.1–1% glutaraldehyde in acetate buffer (100 mL) followed by 2% PF 0.1–1% glutaraldehyde in borate buffer (400 mL). Coronal sections (50 μm) containing the somatosensory thalamus were cut on a vibratome. In the mice, anterogradely labeled axon terminals in POm were revealed with rabbit-anti-PHAL antiserum (1:10 000, 48 h) followed by Cy3-conjugated anti-rabbit IgG (1:500 Jackson ImmunoResearch). PHAL staining and Thy-ChR2 EYFP were examined with a confocal microscope (Olympus, Fluoview FV 1000, ×60/1.35 UPlanSApo, Fluoview 1.6, *x*: 1600–0.132 px/μm; *y*: 1600–0.132 px/μm; *z*: 19–0.71 μm/slice. For bright field examination and correlated light and electron microscopy (EM), BDA was developed with avidin biotinylated horseradish peroxidase complex (ABC in TBS, 2 h, 1:300, Vector Laboratories) using Nickel-intensified diamino-benzidine (DAB-Ni, black precipitate) as a chromogene. Sections containing PHAL-labeled fibers were incubated in the same primary rabbit-anti-PHAL antiserum as above, followed by biotinylated anti-rabbit IgG (1:500, Jackson) and developed with DAB-Ni. To examine the convergence of subcortical and cortical terminals in the thalamus, anterograde tracing from the S1 cortex was combined with vGluT2 immunostaining. Thus, visualization of the tracer with DAB-Ni was followed by incubation with mouse anti-vGluT2 (1:3000, Chemicon, 48 h), then by mouse ImmPRESS, (1:3, Vector, 3 h) and DAB resulting in brown precipitate. For EM analysis, immunostained sections were osmicated, dehydrated, and embedded in Durcupan. Glucose (7%) was added to the osmium solution to preserve color differences. Close apposition of cortical and subcortical terminals were re-embedded, serially sectioned (60 nm), and examined with a Hitachi EM.

### Analysis of Anatomical Data

Photomicrographs were taken with an AxioCam HRC (Carl Zeiss Microimaging, Jena, Germany). Digital montages of serial photos were processed with the “extended depth of field function” of Image-Pro Express 6.0 (Media Cybernetics, Bethesda, MD, USA). When necessary, brightness and contrast were adjusted using Adobe Photoshop CS2 (Adobe Systems, San Jose, CA, USA) applied to whole images only.

To quantify the co-distribution of the 2 large terminal types, we examined the percentage of large cortical terminals (*n* = 1027 terminals, 3 animals) within zones of POm “rich” or “poor” in vGluT2-positive terminals (*n* = 3 animals). vGluT2-rich zones were defined as having more than 15 terminals on the top and bottom surface of the section together, measured within a 100 × 100-µm area (63× oil immersion objective, 1.4 numerical aperture, Supplementary Fig. 1).

To determine the targets of labeled terminals at the EM level, the minor dendritic diameter of random sample or a given target dendrite was measured. The dendritic diameter was the average 3 diameters measured on 3 nonconsecutive EM sections, where the synaptic contacts were established. Statistical significance was assessed by using the Wilcoxon–Mann–Whitney test.

### In Vivo Electrophysiology in Rats

Experiments were performed in adult male rats (250–300 g); *n* = 16, (Sprague Dawley; St-Constant, Québec) and *n* = 24, (Wistar/Charles River, IEM-HAS, Hungary). Rats were anesthetized with ketamine (83 mg/kg) plus xylazine (3.3 mg/kg) and placed in a stereotaxic apparatus. Rats breathed freely and body temperature was kept at 37°C. The deep level of anesthesia was maintained by additional intramuscular doses of anesthetics given at 30 min to 1 h interval (i.m.). The local field potential (LFP) recorded in the barrel cortex (BC) displayed slow oscillations (1–2 Hz) that are characteristic of the stages III-3-4 described by (Friedberg et al. 1999).

Craniotomies were made over the BC and thalamus for insertion of recording electrodes. Cortical LFP was recorded in BC with bipolar tungsten electrode (∼1 MΩ, FHC, Bowdoin, ME, USA) placed 2.5 mm posterior and 2.5 mm lateral to the Bregma and −1.5 mm from the surface of the brain). Signals were filtered (0.1 Hz–5 kHz), amplified (Supertech BioAmp, Supertech, Pécs, Hungary), sampled at 20 kHz (micro 1401 mkii, CED, Cambridge, UK), and recorded by Spike2 5.0 software. For intracellular recordings in the thalamus, electrodes were lowered by a piezoelectric microdrive (Burleigh 6000 ULN or ISS 8200, EXFO, Quebec City, Quebec, Canada) at: 3.3 mm posterior, 2.5 mm lateral to the Bregma for POm, or 3.2 mm lateral for ventral posteromedial nucleus (VPM) and between 4.8 and 6.8 mm from the surface of the brain (Paxinos and Keith 2001). Intracellular recording microelectrodes (∼30 MΩ) were pulled from borosilicate glass capillaries (1.5 mm o.d., 0.75 i.d, Sutter Instrument Co., Novato, CA, USA, or WPI, Inc., Sarasota, FL, USA), filled with a solution of potassium acetate (0.5 M) and Neurobiotin (0.8%, Vector Laboratories). Signals were amplified, filtered (DC-5 kHz, Axoclamp 2B, Axon Instruments/Molecular Devices, Sunnyvale, CA, LinearAmp, Supertech), and sampled at 20 kHz (CED). Neuronal signals were recorded by Spike2 5.0 software. Neuron location was assessed by the intracellular labeling of units.

Cortical spreading depression (CSD) was initiated by a drop of 2 M KCl solution on the exposed cortical surface around the LFP recording electrode in BC.

At the end of the experiments, animals were perfused and the brains were sectioned as above. Neurobiotin was revealed using ABC and DAB-Ni. The sections were either processed for cytochrome oxidase-staining (Veinante et al. 2000) or vesicular glutamate transporter type 2 (vGluT2). vGluT2 immunostaining was used to establish the precise nuclear boundary between VPM and POm. Sections containing labeled neurons were then osmicated, dehydrated, and flat-embedded in Durcupan (Fluka, Buchs, Switzerland) or mounted on gelatin-coated slides, dehydrated, and covered with Depex (AMS, Abingdon, UK) for light microscopical analysis.

### In Vivo Electrophysiology in Mice

Animal preparation and recordings were done with 6–8-week-old Thy1-ChR2 (line 18) (Arenkiel *et al.* 2007) mice anesthetized with 1% isofluorane in O_2_ (SurgiVet Vaporizer). The skull above vibrissal cortex (lateral = 3.0 mm, posterior = 1.1 mm from bregma) was exposed and thinned (for the majority of POm and VPM recordings) or a 0.3 × 0.3-mm craniotomy was made (for BC recordings, dura intact). A craniotomy was made above POm (lateral = 1.25 mm, posterior = 1.7 mm from bregma), and the head was stereotaxically aligned (Wimmer et al. 2004) for precise targeting of POm. All recordings were done in the right hemisphere, at a depth of 0.7 mm from dura mater for L5B-ChR2 and 2.8–3.0 mm from dura mater for POm neurons. In vivo juxtasomal recordings and biocytin fillings were made as described in (Pinault 1996). In brief, 4.5–5.5 MΩ patch pipettes pulled from borosilicate-filamented glass (Hilgenberg, Germany) on a DMZ Universal puller (Zeitz Instruments, Germany) were used, and pipettes were filled with (mM): 135 NaCl, 5.4 KCl, 1.8 CaCl_2_, 1 MgCl_2_, and 5 Hepes, pH adjusted to 7.2 with NaOH, and 20 mg/mL biocytin was added. Bath solution was identical, with the exception of biocytin. Single units were found by the increase of pipette resistance (2–2.5 times the initial resistance) measured in voltage clamp mode. Single-unit recordings were made using an ELC-01X amplifier (NPI Electronics, Germany). Unfiltered and band-pass-filtered signals (high pass: 300 Hz, low pass: 9000 Hz) were digitized at 20 kHz with CED Micro 1401 mkII board and acquired using Spike2 software (both CED, Cambridge, UK). Typically, recordings consisted of one single unit which was filled at the end of the experiment with biocytin using current pulses (Pinault 1996). Whole-neuron voltage recordings in POm were done as described in (Margrie et al. 2002). Pipette solution was (in mM): 130 K-gluconate, 10 HEPES, 10 Na-phosphocreatine, 10 Na-gluconate, 4 ATP-Mg^2+^, 4 NaCl, 0.3 GTP, 0.1 EGTA, 100 µM Spermidine, osmolarity ∼300 and brought to pH 7.2 with KOH. The locations and morphologies of 5 POm neurons that showed strong increase in spiking probability after coincident sensory and cortical stimulation were recovered (not shown).

### In Vivo Laser Stimulation Setup

Stimulation of ChR2 neurons was achieved by a custom-built laser setup consisting of a solid-state laser (Sapphire, Coherent, Dieburg, Germany) with a wavelength of 488 nm and a maximal output power of 20 mW. The sub-millisecond control of laser pulses was achieved by an ultrafast shutter (Uniblitz, Rochester, NY, USA). The laser beam was focused with a collimator into one end of a multimode fiber (Thorlabs, Grünberg, Germany numerical aperture = 0.48, inner diameter = 200 µm). The maximal output power at the end of the fiber was 1 mW, resulting in a maximal power density of 32 mW/mm^2^. Shutter control was implemented with Spike2 software (CED, Cambridge, UK).

### Laser and Whisker Stimulation

Whisker stimulation consisted of 50 ms air puffs (50 mbar) (Ahissar and Zacksenhouse 2001) delivered via a plastic tube with a tube opening of ∼1 mm. The opening was positioned 0.5–2 cm anterior of the stimulated whiskers which were deflected in caudal direction. For POm recordings, the air jet deflected 1 or 2 rows. The latency from command to whisker deflection was measured using 2 methods: First, after each experiment, the air puff was given to a microphone positioned at the same distance as the whiskers and the potential change was read from an oscilloscope. Second, a small magnetic probe (0.5 mg) was glued to one stimulated whisker, and the time of deflection was measured with a custom-built magnetic field detector. The latencies from the command trigger to the potential change as well as to the deflection of the whisker were typically between 22 and 26 ms. Figures and data analysis were corrected for this delay and show the time of whisker deflection. For BC stimulation, the optical fiber was positioned at an angle of ∼86° and with a distance of ∼100 µm to the cortical surface. Laser pulses for the majority of POm and VPM recordings were applied through the thinned skull and for BC recording onto the cortical surface. Laser stimulation consisted of a 5-ms blue laser pulse. The receptive field in vibrissal cortex of a cortically driven POm neuron was estimated by moving the glass fiber in *x-* and *y*-dimensions along the vibrissal field while recording spike responses in POm.

### Analysis of Electrophysiological Data

Data analysis of intracellular recordings in rats was performed with Spike2, Matlab, OriginePro 7.5, and Excel software. EPSPs were discriminated and their rise time (20–80%) was determined with inbuilt functions of Spike2. *Phase analysis:* Raw data were imported to Matlab for analysis using built-in and custom-written functions. LFP was preprocessed using a zero phase-shift low-pass finite impulse response filter with a cutoff frequency of 5 Hz and standardized to zero mean and unitary standard deviation. EPSPs were detected with Spike2 software by applying a slope process (first derivative). The position of the maximum slope of the EPSP was determined and was then used as a reference point to find maximum peak of the EPSP. Double-slope process (second derivative) was used to determine the onset of EPSPs as being the largest rate of change of slope. EPSPs were considered fast “driver-like” if onset to peak was not longer than 1 ms. This measure was based on the findings of (Deschenes et al. 2003) where lemniscal driver EPSP rise times were measured in the VPM of rats under the same anesthetic conditions and recorded with the same method. In our case, EPSP rise times were below the set 1 ms measure similarly to the lemniscal EPSPs (see exact values of 20–80% rise time in the main text). The phases of events (AP of L5 pyramidal neurons or EPSPs in POm neurons) relative to the slow oscillation cycles in the LFP were determined by using the Hilbert transformation of the filtered LFP and taking the angle of the analytic signal at the location of spikes (Hurtado et al. 2004). Slow-wave cycles shorter than 250 ms (corresponding to a 4 Hz upper limit) as well as cycles with LFP amplitudes lower than mean LFP + 2 SD were discarded from the analysis. Phase histograms, mean angles, and mean vector lengths were calculated by the means of circular statistics methods (Fisher 1993; Hurtado et al. 2004).

Extra- and intracellular recordings in mice were analyzed using custom-written software in Matlab (MathWorks, Natick, MA, USA). For both intracellular and extracellular recordings, spike times were extracted by finding local maxima in the temporal derivative of recorded voltage traces (d*V*/d*t*) above a variable threshold (typically 40–50% of maximum d*V*/d*t*). To analyze subthreshold EPSP times in intracellular recordings, we first categorized each repetition of stimulus presentation as “responsive” or “unresponsive” by first normalizing d*V*/d*t* by its maximum and then examining the central portion (between 10th and 90th percentiles) of *P*(d*V*_norm_/d*t*). Compared with trials with EPSPs, *P*(d*V*_norm_/d*t*) was comparably wide for trials with no EPSPs and narrow for trials containing EPSPs; using a threshold in the standard deviation (*σ*) of *P*(d*V*_norm_/d*t*) allowed us to separate “nonresponsive” (high *σ*) and “responsive” (low *σ*) trials. For trials with EPSPs, EPSP events times were found with the same procedure used to find spike times; maximal rate of rise of voltage response at the beginning of an event. Statistical analysis was conducted using Matlab (MathWorks) software. First, datasets of all conditions were tested for normal distribution using the parameter-free 1-sample Kolmogorov–Smirnov test (K–S test). For statistical analysis of the spike probability dependency on the timing of the 2 inputs on single neuron level, the nonparametric *χ*^2^ test was employed. Statistical differences of EPSP amplitudes were analyzed using the Wilcoxon–Mann–Whitney test.

## Results

### Convergence of Large Cortical and Subcortical Terminals in the Thalamus

The posterior nucleus of the thalamus (POm) is known to receive both subcortical (trigeminal) and cortical driver afferents (Hoogland et al. 1991; Lavallee et al. 2005). To test whether driver afferents of cortical and brainstem origin can innervate the same thalamic territories or converge on single thalamocortical cells, we labeled L5 input from the somatosensory cortex by anterograde tracing and the brainstem inputs by type 2 vesicular glutamate transporter immunocytochemistry (vGluT2) a well-established marker of subcortical excitatory inputs (Fremeau et al. 2001; Herzog et al. 2001; Land et al. 2004; Lavallee et al. 2005; Graziano et al. 2008).

**Figure 1.**
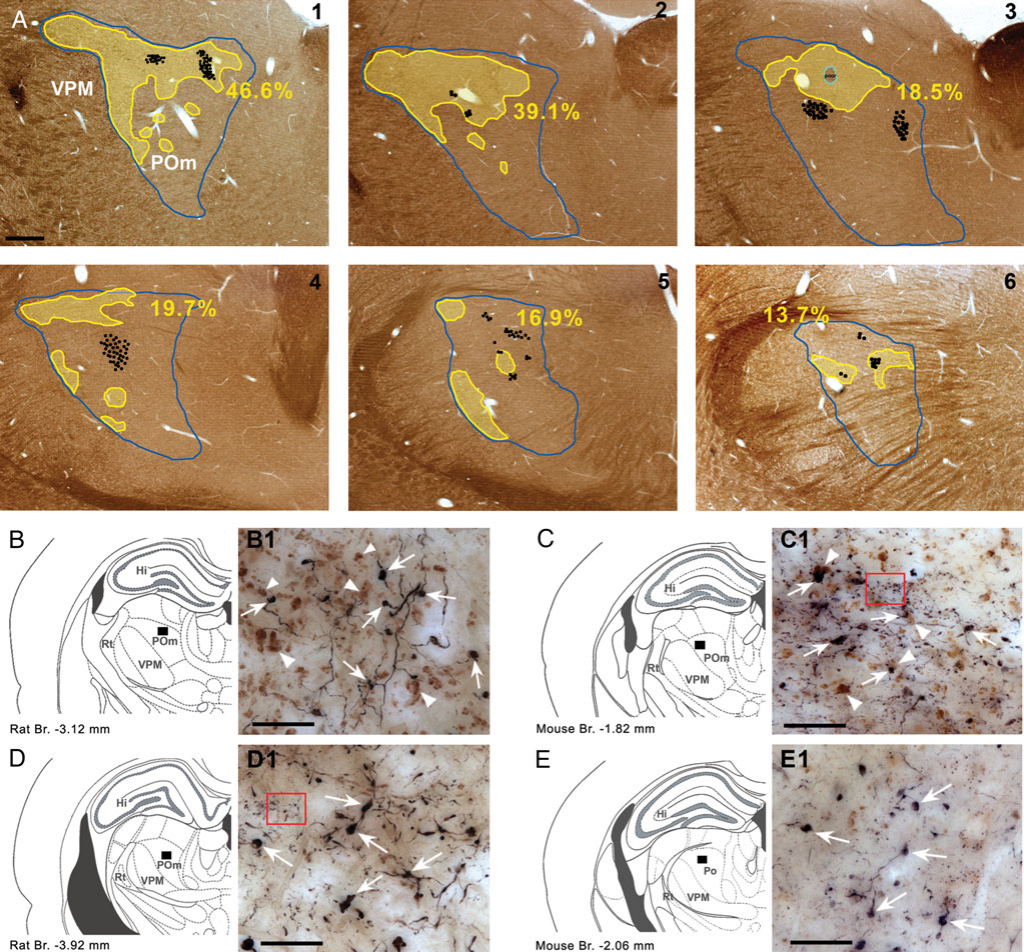
Convergent zones of cortical and subcortical large terminals in POm. (*A*) Distribution of large cortical (black dots; 1 dot = 2 boutons) and subcortical terminals (yellow outline) labeled by anterograde tracing and vGlut2 immunostaining, respectively, in the whole rostro-caudal extent (1–6, rostral to caudal) of rat POm (blue outline) in a representative animal. Yellow outline indicates vGlut2 rich zones. (for definition, see Materials and Methods section). Note the occurrence of large cortical terminals within as well as outside of the vGlut2 rich zones. Percentages represent the fraction of POm area rich in large vGlut2 terminals. (*B* and *C*) Convergent and nonconvergent zones in rat (*B* and *D*) and mouse (*C*–*E*). In *B*–*E*, black rectangles show the position of the high-power light microscopic images (*B*1–*E*1). In convergent zones (*B*1 and *C*1), large PHAL-labeled (black) cortical terminals (arrows) are in close proximity to vGLUT2-immunoreactive (brown) subcortical terminals (arrowheads). In nonconvergent zones (*D*1 and *C*1), only large cortical terminals are visible (arrows) subcortical terminals are absent or occur in low numbers. Note that cortical terminals originating from layer 6 are considerably smaller in size than giant layer 5 boutons (the red framed areas show a group of small PHAL-labeled boutons in *C*1 and *D*1). Scale bar, *A*: 500 μm; *B*1–*D*1: 20 μm.

First, we examined whether subcortical inputs were present in the entire POm since as a recent work in primates (Rovó et al. 2012) indicates large thalamic regions lack vGluT2-positive inputs. The distribution of vGluT2-immunoreactive terminals in POm displayed an inhomogeneous pattern (Fig. 1) in both rats and mice. vGluT2-positive terminals were abundant in rostral POm and were more sparse in caudal POm. Approximately one-fourth to one-third of POm was densely innervated by brainstem terminals (*n* = 3, 27.5% SD: 4.75, Fig. 1*A*, Supplementary Fig. 1), whereas the rest of the POm region had few or no subcortical inputs. Next, we wanted to identify if large cortical inputs targeted vGluT2-rich or vGluT2-poor regions.

**Figure 2.**
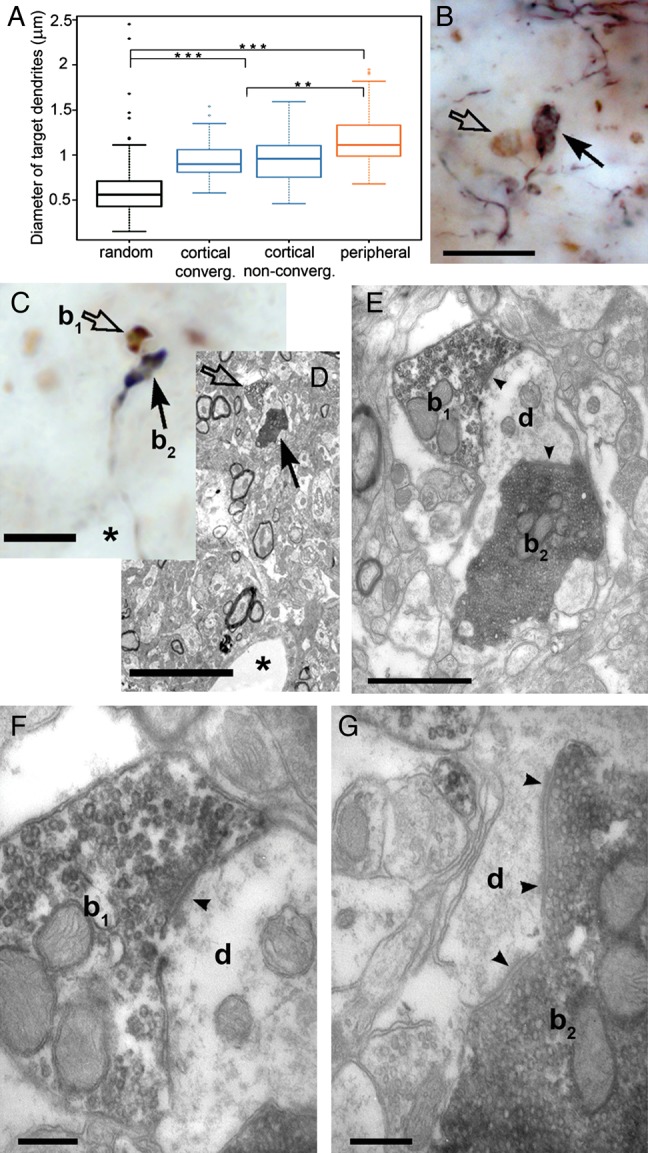
Convergence of cortical and brainstem driver terminals on a single dendrite. (*A*) Whisker plots showing the minor diameter of randomly selected dendrites compared with those postsynaptic to large cortical (in convergent and nonconvergent zones) and subcortical terminals in the rat POm. Both subcortical and cortical drivers innervate large caliber dendrites, which are significantly thicker than the diameter of randomly selected dendrites. (*B* and *C*) A high-power light micrographs of anterogradely labeled large cortical terminals (arrows) and vGluT2-positive subcortical terminals (open arrows) apparently facing a single postsynaptic target in the neuropil of mouse (*B*) and rat (*C*). (*D*) Correlated low-power electron microscopic image of the terminals shown in *C*. A capillary (asterisk) serves as a landmark. (*E*) Electron microscopic images demonstrating that both terminals (subcortical b1, cortical, b2) converge and establish synapses (arrowheads) on the same dendrite (d). (*F*) High-power image of the vGLUT2-positive terminals showing the synaptic cleft (arrowhead) and the presynaptic vesicle accumulation. (*G*) High-power image of the cortical terminal showing additional synapses (arrowheads) on the same target, a characteristic feature of driver terminals. Scale bar, *B*, *C*, *D*: 10 μm; *E*: 1 μm; *F*–*G*: 250 nm.

We injected anterograde tracers into the somatosensory cortex which labeled large and small terminals as descried previously (Hoogland et al. 1991). The distribution of large terminals was comparable to a recent mapping of the S1-POm pathway (Liao et al. 2010). Large cortical terminals were found within POm regions rich in vGluT2 terminals, defining putative zones of convergence (Fig. 1*B*,*C*), as well as in regions having few or no brainstem inputs (Fig. 1*D*,*E***)**. At the electron microscopic level, both cortical and subcortical giant terminals innervated large caliber proximal dendrites, which were significantly thicker than a random selection of dendrites (Fig. 2*A*). This demonstrates that the 2 driver pathways innervate the same dendritic compartment (the proximal dendrite). Within the zones of convergence, giant cortical and giant brainstem terminals were found in close proximity (<5 µm) in both mice and rats (Fig. 2*B*,*C*). In order to verify if close appositions indeed represent convergence on a single target, we performed correlated light and electron microscopy for one double-labeling experiment. This demonstrated that a large cortical and a large brainstem terminal established several asymmetrical synaptic contacts on the same postsynaptic dendrite (Fig. 2*C*–*G*).

### POm Neurons Receive Cortical Driver Signals

Using light and electron microscopy, we demonstrated that driver pathways of different origin can synaptically interact on single thalamocortical cells. However, demonstrating the frequency of convergence is extremely difficult using purely anatomical methods. One should label all cortical and subcortical terminals together with the entire dendritic arbor of POm cells, since converging contacts can occur on distant dendritic branches. The entire procedure should be repeated in different POm sectors containing different number of vGLUT2 terminals on a statistically significant number of neurons, followed by electron microscopic verification of the contacts. Instead, we chose a physiological approach. We defined what percentage of POm neurons receives cortical driver input and thus can potentially receive convergent innervation. To determine this subset of POm neurons, we looked for physiological markers of cortical driver afferents. Driver pathways in the thalamus have been previously characterized by fast-rising, large amplitude EPSPs (Deschenes et al. 2003). In order to identify cortical driver EPSPs, we made intracellular recordings of POm neurons under ketamine/xylazine anesthesia. This anesthesia induces regular slow oscillations consisting of alternating Up states when cortical neurons are active and Down states, when they are silent (Chauvette et al. 2011). We compared the intracellular activity of cells in the POm nucleus, known to receive cortical drivers, to thalamocortical cells of the neighboring VPM, which lacks cortical L5 driver afferents (Hoogland et al. 1991).

**Figure 3.**
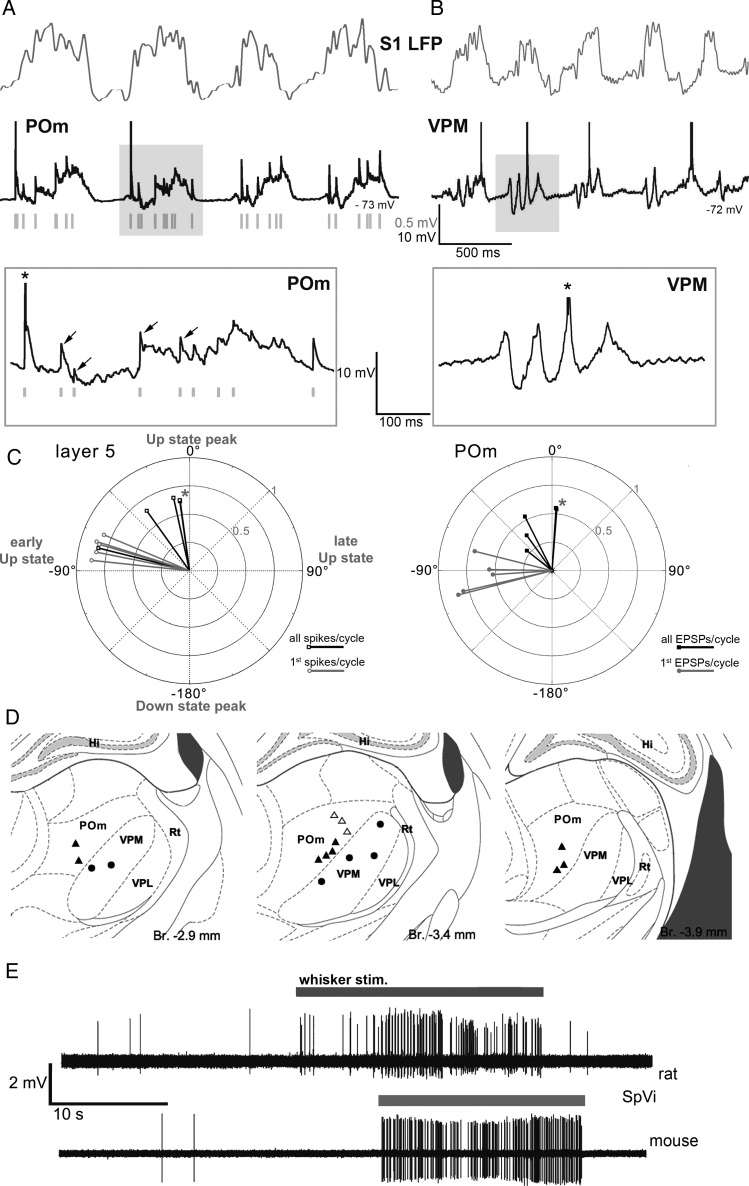
Cortical origin of driver EPSPs in POm cells. (*A* and *B*) In vivo cortical local field potential (LFP, gray) recordings in the rat somatosensory cortex with concurrent intracellular recordings (black) from an ipsilateral POm (*A*) or VPM (*B*) neuron. Intracellular activities during a cortical Up–Down cycle (shaded area) are shown at extended time scale below (boxed). Note fast-rising EPSPs in POm (arrows), appearing at Up-state onset. The intracellular activity of VPM is characterized by rhythmic IPSPs in the spindle frequency range, no fast-rising EPSPs are apparent. Action potentials are truncated (asterisk in the inset); green bars, discriminated EPSPs for the analysis in Figure 3*C*. (*C*) Polar plots of the phase of the action potentials in cortical layer 5 neurons (left) and EPSP onset times in POm neurons (right) relative to the slow cortical oscillation. 360° correspond to one full Up–Down cycle. Zero degree corresponds to the peak of the Up state. Each vector represents the average data of single L5 (right) or POm neurons (left). Black, all events, gray, all first events per cycle. Note that both action potentials in L5 neurons and the fast-rising EPSPs in POm are timed to the early, rising phase of the Up state (negative phase values). Asterisks label 2 cells with almost entirely overlapping phase values. (*D*) Location of recorded and labeled thalamic neurons (triangles: POm) individually identified in POm (filled triangles) and VPM (filled circles). (*E*) Spontaneous and whisker stimulation evoked activity of SpVi neurons in the rat and mouse. Juxtacellular recordings from SpVi neurons in anesthetized animals. The baseline activity of SpVi cells is characterized by very sparse background firing and with brisk response to whisker stimulation (gray line) with a hand held probe.

Intracellular recordings of rat POm neurons (*n* = 15) revealed a barrage of fast-rising (0.26 ± 0.06 ms, quantified in 9 cells) large amplitude EPSPs (Fig. 3). The EPSPs were similar in size and shape to those evoked by driver inputs described in earlier in vitro and in vivo studies (Deschenes et al. 2003; Groh et al. 2008). The EPSPs were rhythmically coupled to the cortical slow oscillation and occurred during the Up states when cortical neurons are active (Fig. 3*A*) suggesting a cortical origin for these EPSPs. In contrast to POm neurons, none of the VPM neurons (*n* = 8, Fig. 3*B*) displayed large fast-rising EPSPs during the slow oscillation. The positions of 12 POm cells and the 6 VPM cells which were unequivocally localized (individually identified: POm = 9, VPM = 6) are shown in Figure 3*D*.

**Figure 4.**
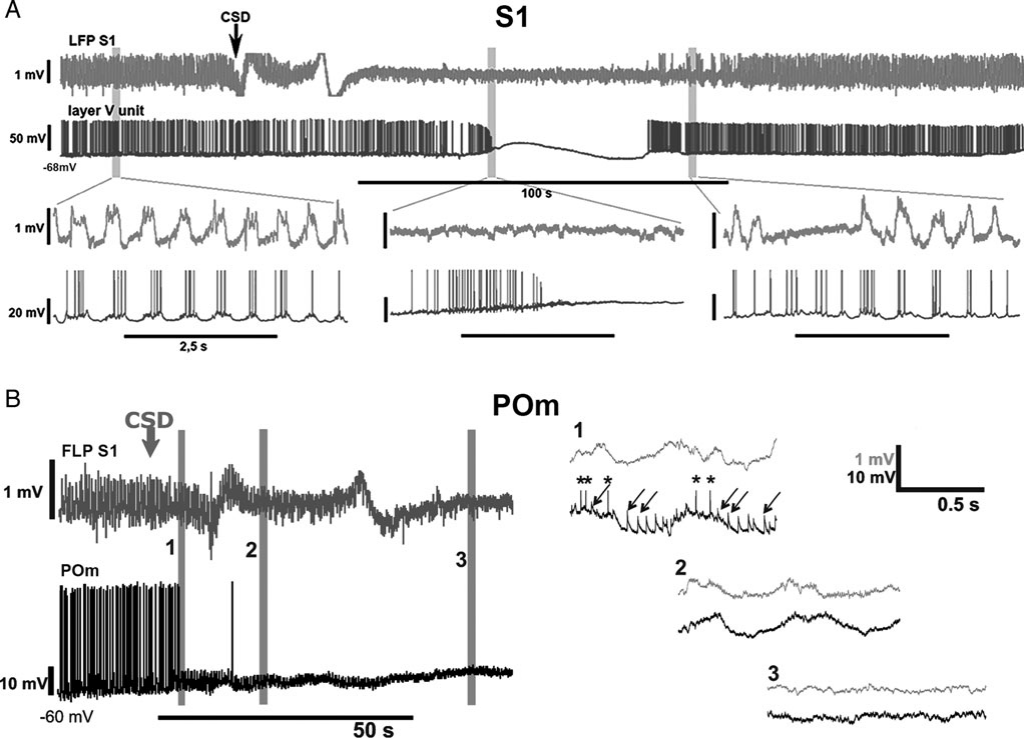
Response of intracellular L5 and POm activity to the altered cortical activity. (*A*) Cortical LFP (top row, light gray) and intracellular activity of a rat L5 cortical neuron (bottom row, dark gray) is shown before, during and after a cortical spreading depression (arrow at CSD onset) induced by 2 M KCl in the primary somatosensory cortex (BC). Periods indicated by gray bars are shown below on an extended time scale. Before the CSD (left) L5 neurons display rhythmic spiking activity and depolarizing membrane potential oscillation locked to the Up states of the LFP. During the CSD (middle), the rhythmic L5 activity is first replaced by a ramp depolarization and high-frequency tonic firing (depolarizing phase) then by a depolarization blockade and a complete cessation of firing. After the CSD (right), L5 activity resumes. The time difference between the onset of CSD in the LFP and in the intracellular activity is the result of the propagation of spreading depression from the site of induction (LFP recording electrode) to the site of intracellular recording. (*B*) Cortical LFP (top row, gray) and intracellular activity of a POm neuron (bottom row, black) before and during CSD induced in the primary somatosensory cortex (S1). Periods indicated by gray bars (1–3) are shown on an extended time scale on the right. During the depolarization phase of the CSD, when L5 neurons display high-frequency tonic firing (see *A*) the POm neuron receives a barrage of high-frequency EPSPs (1). In the next phase of the CSD (2–3), when L5 neurons stop firing, all EPSPs disappear in POm.

Next, we performed a series of experiments to demonstrate the cortical origin of the EPSPs recorded in POm neurons. First, to test if these transients may arise from subcortical sources, we recorded neuronal activity in the spinal trigeminal nucleus (SpV), the origin of subcortical input to POm (Veinante et al. 2000). Spontaneous neuronal activity in SpV of rats and mice was negligible under our recording conditions (rat, *n* = 30 neurons, Fig. 3*E*, mouse, *n* = 54 cells, not shown), SpV is therefore unlikely to be the source of EPSPs in POm. In contrast, recording the spiking activity of L5 pyramidal neurons (*n* = 25), demonstrated that L5 spiking was rhythmically coupled to the cortical slow oscillation. We used the cortical Up- and Down-state cycle as a temporal reference to study the timing between L5 spikes (*n* = 5) and large EPSPs in POm (*n* = 5). Spikes in L5 pyramidal neurons were locked to the same early phase of the Up state as the EPSPs of the POm neurons (Fig. 3*C*). Finally, we altered the cortical activity by applying KCl and induced CSD while recording EPSPs in POm (Fig. 4). In the initial phase of the CSD, when cortical activity is enhanced (Fig. 4*A*), the frequency of POm EPSPs increased and became arrhythmic (Fig. 4*B*, trace 1). In the next phase of the CSD, when cortical activity was blocked, the fast-rising EPSPs disappeared in POm (Fig. 4*B*, trace 2, 3). The EPSPs resumed after recovery from CSD.

These data demonstrate that the L5–POm synaptic activity can be detected during spontaneous network activity as fast-rising EPSPs. Since fast-rising EPSPs were found in all recorded POm neurons located at various places within POm (Fig. 3*D*), we conclude that most if not all POm neurons receive L5 input. As a consequence, the entire territory receiving vGluT2 immunoreactive terminals (Fig. 1) can be treated as convergent zone of cortical and brainstem drivers in POm.

### Functional Interaction Among the Cortical and the Brainstem Driver Pathways

**Figure 5.**
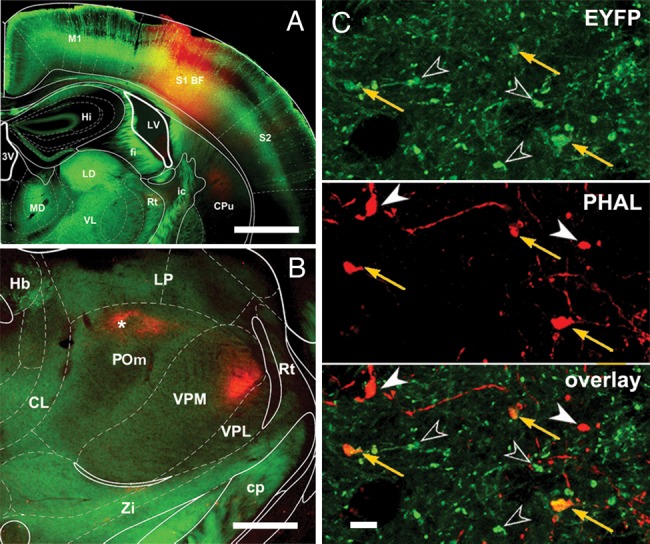
ChR2-EYFP-positive large cortical terminals from barrel cortex innervate POm. (*A*) Injection site of the anterograde tracer PHAL into the mouse somatosensory cortex. (*B*) Low-power image of the anterogradely labeled PHAL-containing cortical fibers in the thalamus. Asterisk denotes the location of high-power images (*C*). (*C*) Large ChR2-EYFP-positive terminals (arrows and open arrowheads, top) co-localize with PHAL-labeled terminals (orange arrows, middle and bottom). As EYFP is expressed only in layer 5 cells, the co-localization demonstrates that ChR2-positive cortical L5 neurons project to POm with large driver terminals. Open arrowheads, EYFP-positive, PHAL-negative terminals; filled arrowheads, EYFP-negative, PHAL-positive terminals. Scale bar, *A*: 1 mm; *B*: 500 μm; *C*: 5 μm.

For selective activation of cortical L5 and subcortical drivers, we used transgenic mice. Control of the cortical driver pathway was achieved by an optogenetic approach (Boyden et al. 2005), using the Thy1-ChR2 transgenic mouse line in which channelrhodopsin-2 (ChR2) is expressed in the cortex exclusively by L5 pyramidal neurons (Arenkiel *et al.* 2007; Stroh *et al.* 2013). Anterograde tracing from the somatosensory cortex directly confirmed the presence of ChR2-positive giant terminals in the POm originating from cortical L5 ChR2-positive neurons (Fig. 5), thus allowing the activation of the corticothalamic driver pathway. The subcortical pathway was activated by whisker stimulation (Trageser and Keller 2004; Lavallee *et al.* 2005). To test functional interaction between the 2 pathways, we tested whether POm spiking probability changed when the cortical driver pathway was co-stimulated for a range of time intervals relative to whisker stimulation.

**Figure 6.**
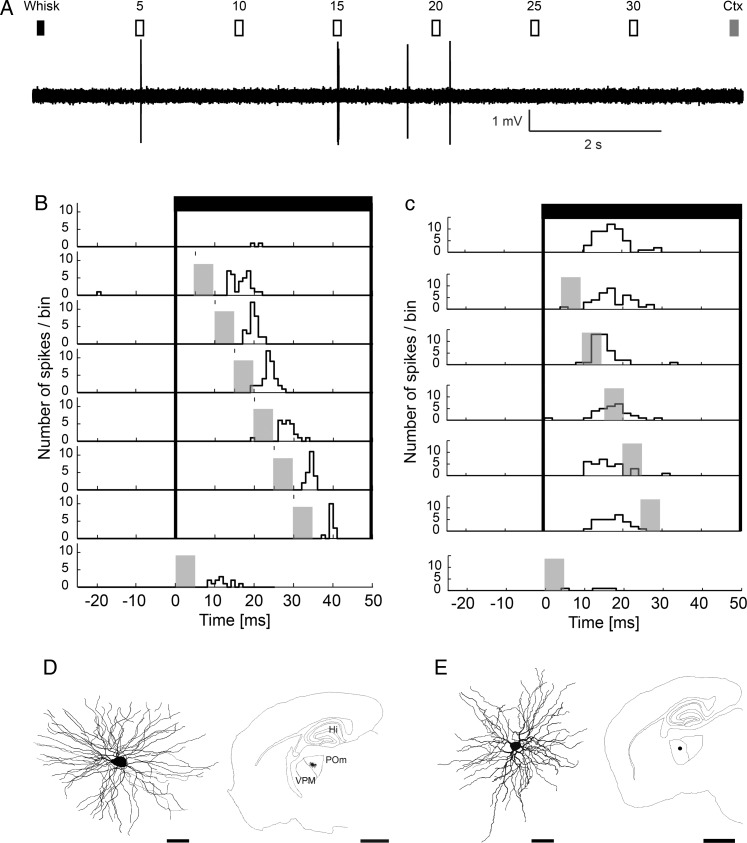
Thalamic integration of cortical and brainstem drivers in POm but not in VPM. (*A*) Stimulation protocol and recording of a mouse POm driver integrator neuron in response to sensory stimulation (Whisk, black rectangle), cortical stimulation (Ctx, gray rectangle), or paired stimuli (open rectangles) with different delays (5–30 ms). Individual trials began with a whisker deflection alone and were followed by combined whisker and BC stimulations with different delays as indicated. Trials ended with BC laser stimulation. (*B*) Peristimulus time histogram (PSTH) for each stimulus condition in a POm cell. Whisker stimulation, black bars (*t* = 0 ms); BC stimulation, gray bars (*t* = 5–30 ms). Note low response magnitude to whisker alone (top row) and cortex alone (bottom row) conditions and a prominent increase of firing rate with paired stimulation (6 middle rows) with different delays. (*C*) VPM neuron responded well to whisker deflection (black bars) but this response was not enhanced with additional BC stimulation (gray bars). (*D*) Neurolucida reconstruction (left) and position (right) of a POm neuron displaying driver integration in a parasagittal section. (*E*) Neurolucida reconstruction (left) and position (right) of the VPM neuron in a parasagittal section. The activity of the cell is shown in *C*. Note the different size of the scale bars. Scale bar, cells: 20 μm; slice outline: 1 mm.

POm cells responded to high laser intensity with high probability of firing, which was not useful to detect the interaction due to a ceiling effect. In order to lower the probability of POm response to cortical stimulation, the intensity of the photostimulus was reduced below 32 mW/mm^2^, thereby reducing the probability of evoked spikes in POm to 10 ± 8% (Fig. 6*A*,*B*). The response of POm neurons to whisker stimulation is known to be very low, since it is under strong feed-forward inhibitory control, as described before in the rat (Trageser and Keller 2004; Bokor et al. 2005; Lavallee et al. 2005). Confirming these data in mice, we found that POm neurons responded with low spiking probability after whisker stimulation (Fig. 6*A*,*B*), which was useful for a pairing protocol.

In the first set of experiments for the combined cortical and sensory stimulation, whisker deflections were followed by the laser pulses at different latencies (5–30 ms, at 5 ms increments, Fig. 6*A*). We found that spiking probability increased significantly for the paired stimuli compared with the individual stimuli in 10 of 14 recorded POm neurons (Fig. 6*B*). The increase in spiking probability after paired stimulation was supralinear with a ∼16-fold and ∼4-fold increase compared with whisker or L5 stimulation, respectively. Responses to paired stimulation were ∼2.7-fold larger compared with the sum of cortex and whisker responses (*n* = 10). In contrast, paired stimulation did not alter whisker responses in VPM (Fig. 6*C*). Labeling and reconstruction of the recorded thalamic neurons revealed typical multipolar thalamocortical cells (Fig. 6*D*,*E*).

**Figure 7.**
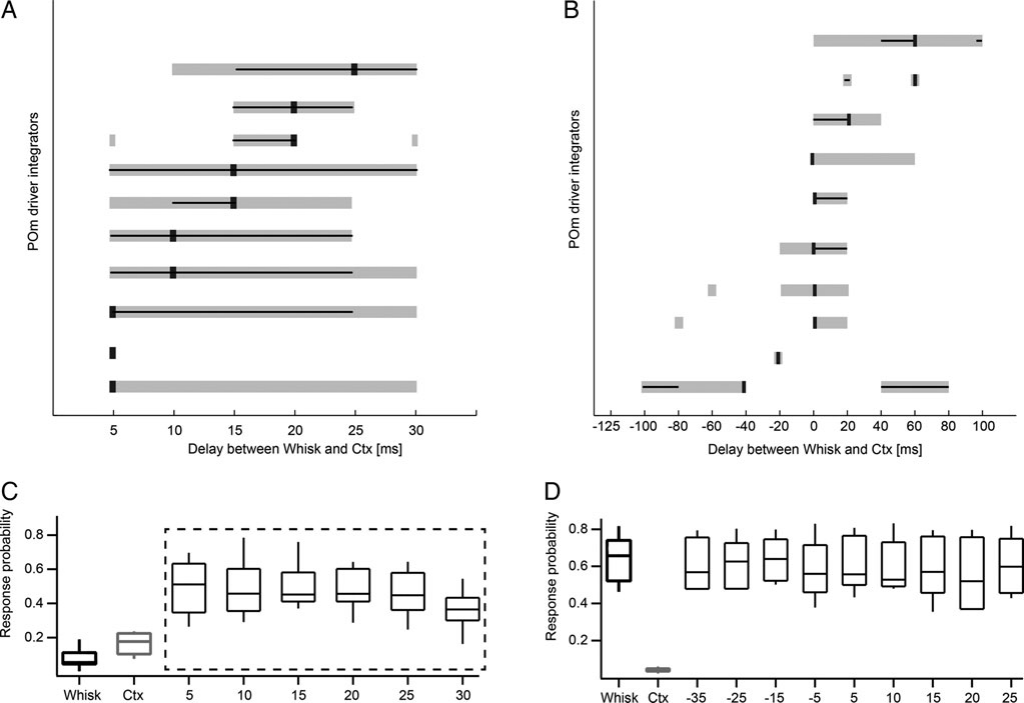
Temporal tuning of individual POm neurons. (*A*) Temporal tuning of 10 POm mouse driver integrators with short delays. The delays that evoked the largest response are indicated by black squares. Neurons were sorted according to their peak tuning, with neurons tuned for long delays on top. The width of the integration window (gray bar) indicates the delays that evoked significantly greater responses than the linear sum of whisker (Whisk) and cortical (Ctx) stimulation (*P* < 0.001, *χ*^2^ test). Black lines span the delays at which responses were greater than the linear sum of whisker (Whisk) and cortical (Ctx) stimulation and did not significantly differ from those at the delays of the largest response (tuning precision, *P* > 0.05, *χ*^2^ test). Longer lines indicate less precise tuning. (*B*) Temporal tuning of 10 driver integrators which were tested for longer delays and reverse order (labeling as in *A*). Negative delays indicate that L5B (Ctx) preceded whisker (Whisk) stimulation; positive delays indicate that whisker stimulation preceded L5B stimulation. Note asymmetrical tuning (i.e., effective integration) when whisker stimulation precedes cortical stimulation in the majority on neurons. (*C*) Average spike probabilities from 10 driver integrators tested with short delays between whisker and cortex stimulation. The response was largest when the whisker stimulus was followed by the laser stimulus with a delay of 5 ms. This condition was compared with all other conditions by employing the nonparametric *χ*^2^ test. The average peak response (5-ms delay condition) was not statistically different from the other paired stimuli responses (tuning precision *P* > 0.05, *χ*^2^ test, dashed box) and all paired stimuli responses evoked a significantly larger response than the sum of Whisk and Ctx (tuning width *P* < 0.001, *χ*^2^ test, dashed box). (*D*) In VPM cells, combined stimulation did not result in statistically different responses compared with whisker stimulation alone.

The magnitude of increase in POm was dependent on the recorded neuron, but more importantly on the delay between whisker and cortical stimulation (Figs 6*B* and 7*A*). Temporal tuning of 10 driver integrators was analyzed for paired stimulation with different latencies (5–30 ms, Fig. 6*A*). We characterized the temporal tuning for each neuron using 3 parameters: 1) the delay between the 2 stimuli that evoked the largest response (tuning peak), 2) the range of delays that evoked significantly larger responses than the sum of L5B and whisker stimulation (tuning width), as well as 3) the delays that evoked responses statistically indistinguishable from the tuning peak (tuning precision). The delays that evoked the largest response were variable across neurons (peak response, black square in Fig. 7*A*). To estimate the precision of tuning, the peak response for each neuron was tested for statistical difference from all other responses. Delays that evoked statistically identical responses as the peak response (*P* > 0.05, *χ*^2^ test) were also variable across neurons (black lines in Fig. 7*A*). The width of the integration window (delays that evoked significantly greater responses than the sum of whisker and cortex stimulation) is indicated with gray bars in Figure 7*A*. In general, the temporal tuning parameters displayed large individual variability among POm neurons (Fig. 7*A*). As a result, pooling data across the population averaged out statistical differences of the temporal tuning, such that the average tuning width and precision spanned all tested latencies (Fig. 7*C*, dashed box, tuning width *P* < 0.001, tuning precision *P* > 0.05, *χ*^2^ test). In contrast, in VPM cells, combined stimulation did not result in statistically different responses compared with whisker stimulation alone (Fig. 7*D*).

In order to estimate the maximal expansion and distributions of tuning windows in a different set of 10 POm integrator neurons, the integration time windows were tested for longer delays and in reverse order between L5B photostimulation and whisker stimulation (−125 to –100 ms, 20 ms increments) (Fig. 7*B*). Tuning parameters were again variable across POm neurons with peak responses ranging from −40 to 60 ms (mean 8 ± 31.6 ms) and tuning precision ranging from 20 to 80 ms (mean 28 ± 19.3 ms) and tuning width ranging from 20 to 100 ms (mean 50 ± 30.2 ms). The integration windows spanned a large range of delays from −100 to +100 ms (Fig. 7*B*) and statistically significant responses could be evoked below the delay range tested in 9 of 10 cells. The temporal tuning had the tendency to be asymmetrical, such that most neurons preferably responded when the whisker stimulus preceded the L5B stimulus.

Taken together, these data suggest that a range of stimulus latencies are coded by individual POm neurons via supralinear summation of cortical and whisker driver signals.

### Intracellular Correlates of Driver Integration

**Figure 8.**
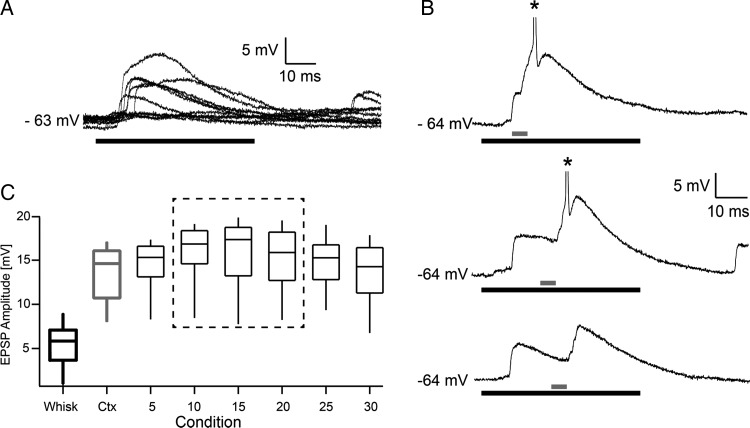
Integration of cortical and peripheral EPSPs by POm neurons. (*A*) EPSP responses to whisker stimulation (black line) in mouse. (*B*) EPSP responses to combined stimuli (gray: barrel cortex stimulation, black: whisker stimulation). Delays from top- to bottom panel = 10, 20, 25 ms. (*C*) Box Plot of EPSP peak amplitudes for each condition (x-axis). Only the EPSPs that did not trigger spikes were selected. EPSP peak amplitudes were largest when the whisker stimulus was followed by the laser stimulus with a delay of 15 ms. This condition was compared with all other conditions using the nonparametric Wilcoxon–Mann–Whitney test. Statistically indistinguishable peak amplitudes (*P* > 0.05 for 10, 15, and 20 ms delays) are indicated by the dashed box and resemble the delay tuning for this neuron. All other conditions were statistically different from the 15 ms delay condition with *P* values < 0.035.

To investigate the underlying mechanisms for the integration of cortical driver output and sensory brainstem signals, we made whole-cell recordings of subthreshold responses in POm to the same stimulation paradigms. Using high laser intensities (32 mW/mm^2^), the initial response was a short latency (6.7 ms ± 1.37), fast- rising (∼1 ms), and large amplitude (13.4 ± 4.2 mV) EPSP, which triggered single spikes or bursts. Lower intensities evoked EPSPs with similar onset latency and spikes with lower probability and longer latency (not shown). Whisker-evoked EPSPs had more variable amplitudes and onsets (9.13 ± 2.11 ms, *n* = 6, Fig. 8*A*) and elicited spikes in POm neurons in only a minority of experiments (6 of 33 neurons, extra and intracellular recordings together). Experiments with the paired stimulation paradigm suggested that the time course of the cortical EPSP provides a time window for summation with whisker-evoked EPSPs (Fig. 8*B*). The probability that this summation was large enough to reach spike threshold depended on the timing of the 2 stimuli (examples for delays = 10, 20, 25 ms in Fig. 8*C*). We addressed the properties of summation by comparing EPSP peak amplitudes for the different stimulus conditions (cortex, whisker, paired, Fig. 8*C*). The sum of EPSP_Ctx_ (13.4 ± 3.6 mV) and EPSP_Whisk_ (5.5 ± 3.0 mV) is larger than EPSP_Paired_ (15.6 ± 4.4 mV), indicating sublinear summation of EPSPs. EPSP summation is expected to be sublinear if closely spaced synapses are evoked by synchronous activation (Tamás et al. 2002). However, there is also a technical reason for this sublinearity. To estimate the peaks of the EPSPs, we analyzed only EPSPs that did not evoke spikes and thus underestimated the real size for the large EPSPs which were evoked by the paired stimulation.

In summary, the integration of EPSPs originating from the brainstem and the cortical driver pathways led to a pronounced supralinear increase in spike responses in POm (Figs 6 and 7), due to the nonlinear process of spike initiation.

## Discussion

**Figure 9.**
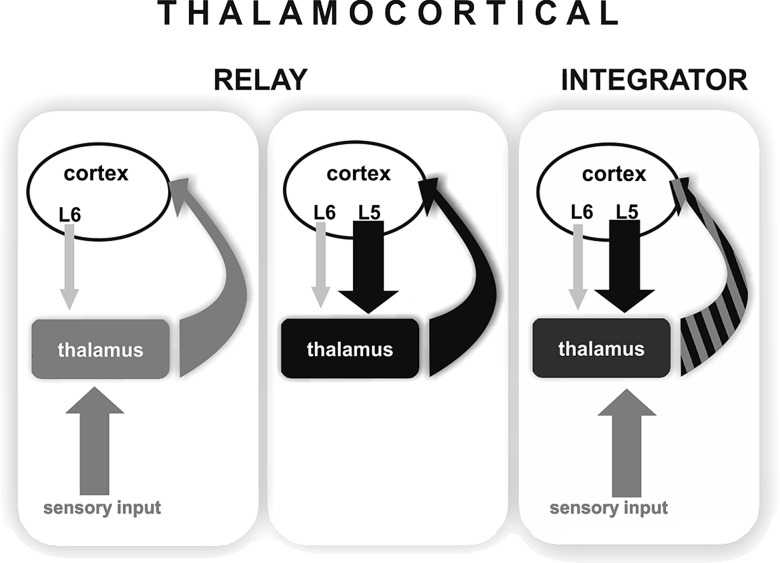
Modes of operation in the thalamocortical system. (Left) Sensory drive. Thalamus faithfully relays sensory information (gray arrow) to the cortex (curved gray arrow). Thalamic activity is determined by the subcortical driver input while cortex modulates sensory transfer via weak layer 6 synapses (thin light gray arrow). (Middle) Cortical drive. Strong cortical signals from layer 5 (black arrow) drives thalamocortical activity which is fed back to cortical circuits (curved black arrow). (Right) Integration of convergent driver inputs. A single thalamocortical neuron receives convergent driving inputs originating from subcortical (gray arrow) and cortical (black arrow) sources. The output (curved black–gray striped arrow) represents the integrated driver activity arising from different sources. Note that the cortical influence on thalamus is qualitatively different in the first and third conditions. In case of sensory drive, layer 6 only modulates the ascending information whereas in case of driver integration, a strong instructive cortical signal summates with sensory information.

We report here the convergence of cortical and subcortical drivers on the same thalamic neurons. Via this connection, the cortical L5 output is able to exert powerful control on the transfer of incoming sensory information in the thalamus. Integration of drivers of different origins has been unknown in the thalamus. Based on these data, we propose that information transfer in thalamocortical neurons can be carried out, not only in the relay mode as was previously known, but also in an integrator mode when the messages of 2 comparably strong inputs of different origin are combined (Fig. 9).

### Integration of Cortical and Subcortical Drivers

Our data show that L5 corticothalamic signals directly interact with incoming sensory information and dramatically alter the output of thalamocortical cells. It should be emphasized that the interaction between descending cortical L5 information and ascending sensory messages in the thalamus is qualitatively different compared with the interaction of cortical layer 6 pathway with ascending inputs. Layer 6 terminals in the thalamus are small, innervate distal dendrites via single synapses, and do not represent a driver input (Reichova and Sherman 2004). Furthermore, collaterals of layer 6 fibers contact the reticular thalamic nucleus and can thereby exert feed-forward inhibition on the thalamocortical cells. Thus, L6 can suppress sensory responses via disynaptic inhibition (Olsen et al. 2012). In contrast, activation of the L5 corticothalamic pathway never suppressed sensory responses; instead, the powerful descending cortical L5 input supralinearly increased ascending sensory responses within a well-defined time window. The patch-clamp recordings (Fig. 8*C*) revealed fast, large amplitude EPSPs following both the cortical and the subcortical stimulation in one relay cell. Direct comparison of the effects of paired stimulation on EPSP size and spike probabilities (Fig. 8*C* vs. Fig. 7*C*) indicates a difference in summation. Spike probabilities summate in a supralinear fashion (almost 3-fold increase), while EPSPs do not. The translation of EPSPs into spikes is a highly nonlinear process which is particularly strong when large EPSPs drive the cell close to the AP threshold, which is true for the driver inputs that we recorded.

Previously, it was unknown what percentage of thalamocortical cells in a higher order nucleus (like POm) is innervated by L5 afferents. In this study, we identified fast-rising driver EPSPs in all POm cells which appeared at the same phase of the cortical oscillation as L5 firing. Their frequency increased to cortical activation and they disappeared after cortical inactivation. They were present in all POm neurons but absent in all VPM neurons which are known to lack L5 input. This suggests that most if not all POm neurons receive L5 input. As a consequence, all POm neurons receiving subcortical (vGluT2-positive) inputs can be regarded as driver integrator neurons which are controlled by near coincident activity of cortical L5 and subcortical driver messages.

The present morphological and physiological data together indicate that the site of supralinear signal summation is the thalamus. In theory, summation of the sensory and cortical signals may take place in the cortex or in the brainstem and then projected to the thalamus. According to this scheme, increased afferent activity, not the convergence of drivers, would lead to the measured increase in thalamic output. Our present data do not support these alternative scenarios. First, morphological data show the convergence of drivers in the thalamus. Second, increased thalamic output cannot be the result of increased brainstem activity, because most POm neurons were asymmetrically tuned and responded only when whisker stimulation was followed by L5 stimulation. As a consequence, the ascending sensory information already passes the trigeminal relay by the time cortical input arrives to the brainstem and would have the ability to affect trigeminal output. Third, the temporal tuning of POm cells indicates that cortical integration is also unlikely due to time constraints. Half of the integrator neurons (10 of 20) had tuning peaks between 0 and 10 ms delay. For the 10 neurons tested with the short-delay protocol allowing better temporal resolution (Fig. 7*C*), the average peak response was at the 5 ms condition. This short delay indicates that sensory information has no time to reach the cortex and alter the response magnitude or probability of L5 neurons to laser stimulation. Finally, intracellular recording demonstrated the integration of 2 distinct EPSPs an early EPSP with a latency of trigeminal inputs and second EPSP linked to the cortical laser stimulation (Fig. 8). Cortical L5 integration of the VPM input with laser stimulation would have resulted in increased cortical EPSPs, without trigeminal EPSP, which we did not observe. Thus, the physiological data demonstrate that cortical or brainstem summation is not a possible scenario and instead strongly suggests that cortical and sensory signals are summated in the thalamus, which is supported by the anatomical data as well.

Polysynaptic activation via the major S1 output regions (basal ganglia, M1 or zona incerta) can also be refuted (see Supplementary Material) in favor of the more plausible monosynaptic pathway.

### Temporal Integration Window

We observed that the integration of cortical and subcortical signals is temporally tuned. Interestingly, individual thalamic neurons are differently tuned to the timing between sensory and cortical activation, raising the possibility that these timing differences are spatially mapped in the thalamus. The integration windows were quite long and spanned tens of milliseconds (Fig. 7). The length of the integration window is likely due to the slow timescale of the whisker- and cortex-evoked EPSPs (Fig. 8*A*,*B*). The EPSP timescale is mostly determined by intrinsic (postsynaptic) dendritic properties, including T-type calcium channels, which cause long, plateau-like depolarizations, typical for thalamic neurons (Jahnsen and Llinas 1984). In this context, it should be emphasized that the present experiments were performed under anesthesia, when thalamic neurons are more hyperpolarized then during the awake state. As the availability of T-type calcium channels is dependent on the previous history of the membrane potential (Jahnsen and Llinas 1984; Tscherter et al. 2011), the integration window of POm cells may be different during active whisking and may be dynamically modulated by ongoing behavioral context.

Response magnitude increased when cortical stimulation preceded whisker stimulation, but not the other way around. Further studies are needed to examine if this is a real asymmetry, which has network basis and not because of the different type (laser vs. puff) or length (5 vs. 50 ms) of the stimulation used here.

### Functional Implications

Earlier studies demonstrated that both the L5 and trigeminal pathways are driver inputs in POm (Hoogland et al. 1991; Williams et al. 1994; Reichova and Sherman 2004; Lavallee et al. 2005; Groh et al. 2008), thus the question arises why 2 powerful pathways should interact. One peculiarity of the subcortical drive in POm is that its activation by passive whisker stimulation is typically not sufficient to evoke postsynaptic spikes, which is confirmed by our present data. The low response magnitude of POm cells is the consequence of a strong subcortical feed-forward inhibition which is able to suppress sensory thalamic responses (Bartho et al. 2002; Trageser and Keller 2004; Bokor et al. 2005; Lavallee et al. 2005). Our present data demonstrate that coincident activity of converging cortical and subcortical drivers can overcome this inhibitory gate within a well-defined time window. According to this scheme, sensory transmission in POm is contingent upon ongoing cortical L5 activity and the relative timing of sensory and cortical events (an “AND-gate” function). In behaving animals, cortical and trigeminal driving inputs can simultaneously arrive in POm, for example, when an animal explores its environment by active whisking. In this case, both the subcortical and cortical drivers carry sensory, motor, or proprioceptive signals which can be integrated in the thalamus.

Earlier theoretical and experimental studies have already proposed that POm neurons can perform such an AND-gate function (Ahissar 1998; Ahissar et al. 2000). According to these studies, POm neurons can transform the temporal code of the sensory organs to a rate code used by cortical networks with the help of a strong cortical feedback signal. It was also suggested that during the later phases of repetitive whisking bouts POm neurons switch from a relay mode to the AND-gate mode. In this mode, POm neurons are active only when their 2 major inputs, from the brainstem and cortex are co-active. (Sosnik et al. 2001). Integration of sensory and cortical drivers by single POm neurons, as demonstrated in this study, supports this concept. The dynamics of this circuit should be further examined in future experiments by entraining the system at whisking-like frequencies (5–11 Hz). Given that cortical and brainstem giant synapses in the thalamus undergo strong adaptation (Ahissar et al. 2000; Deschenes et al. 2003; Reichova and Sherman 2004; Groh et al. 2008), repetitive stimulation decreases the spike probability. Hence, coincident inputs may become even more important to reach AP threshold during consecutive stimulation.

In summary, the present data demonstrate that the cortical layer 5 has a powerful control over ascending sensory information at the level of the thalamus. The converging information channels described here show that an invariant environmental cue (in this case a sensory stimulus) can be fundamentally changed in the thalamus by descending cortical signals and thus perceived differently by the cortex. The results also show that individual thalamocortical neurons do not always relay a single type of message but can integrate powerful signals of different origin (Fig. 9). Thalamocortical neurons with dual driver inputs work as integrators when both driver inputs are active within a well-defined time window.

Considering the anatomical evidence for L5 giant terminals in portions of the auditory, visual, somatosensory, and motor thalamus across species (Rouiller and Welker 2000), driver convergence may be a general feature in the thalamus. Indeed, recently, zones of convergence have also been identified in the macaque thalamus showing its phylogenetically conserved nature (Rovó et al. 2012).

## Supplementary Material

Supplementary material can be found at: http://www.cercor.oxfordjournals.org/.

## Authors’ Contributions

L.A., A.G., H.B., and M.D., designed the experiments, A.S. and A.G. established the photo-stimulation setup, H.B. and A.G. performed physiological experiments, V.P. performed morphological experiments, H.B., B.H., A.G., and R.A.M. analyzed the data; L.A., H.B., A.G., and R.A.M. wrote the manuscript with the contribution of all authors.

## Funding

The experiments in Munich were funded by the DFG Sachbeihilfe (GR 3757/1-1), the Institute of Advanced Studies of the Technische Universität München and the Max-Planck Society. Experiments done in the IEM HAS were supported by the Wellcome Trust (L.A. is in receipt of a Wellcome Project Grant), the Hungarian Scientific Research Fund (Grants OTKA T 75676 and OTKA K 81357), and the National Office for Research and Technology-French National Research Agency TéT Fund (NKTH-Neurogen). Funding to pay the Open Access publication charges for this article was provided by The Wellcome Trust.

## Supplementary Material

Supplementary Data
