# Supplementary material for: Convergence of Cortical and Sensory Driver Inputs on Single Thalamocortical Cells
Source: Cereb Cortex. 2013 Jul 3;24(12):3167–79. doi: 10.1093/cercor/bht173 (PMC4224239; doi:10.1093/cercor/bht173)
Supplement: Supplementary Data [file supp_24_12_3167__index.html]

Convergence of Cortical and Sensory Driver Inputs on Single Thalamocortical Cells — Convergence of Cortical and Sensory Driver Inputs on Single Thalamocortical Cells — Supplementary Data 

# Convergence of Cortical and Sensory Driver Inputs on Single Thalamocortical Cells

## 

Supplementary Data

**Files in this Data Supplement:**

- Supplementary Data - Pdf file
