# Supplementary material for: Convergence of Cortical and Sensory Driver Inputs on Single Thalamocortical Cells
Source: Cereb Cortex. 2013 Jul 3;24(12):3167–79. doi: 10.1093/cercor/bht173 (PMC4224239; doi:10.1093/cercor/bht173)
Supplement: Supplementary Data [file supp_bht173_bht173supp.pdf]

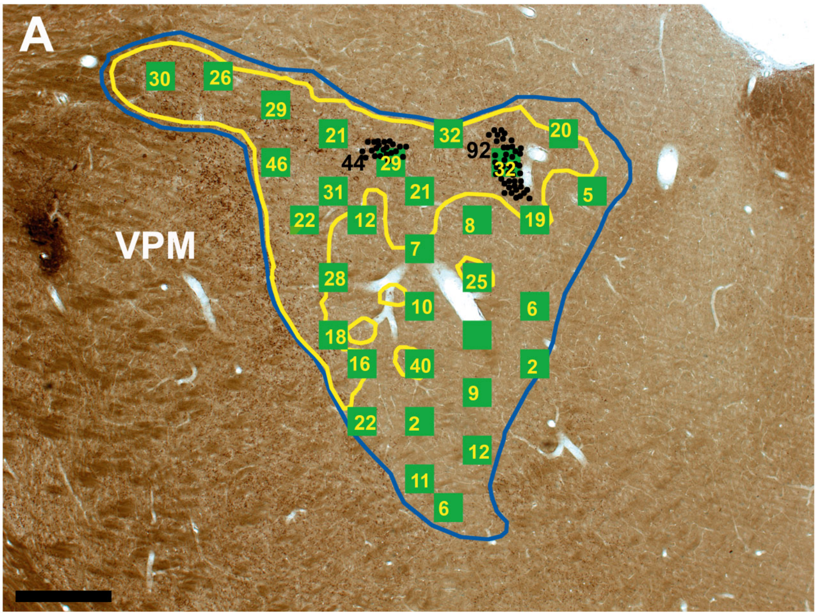

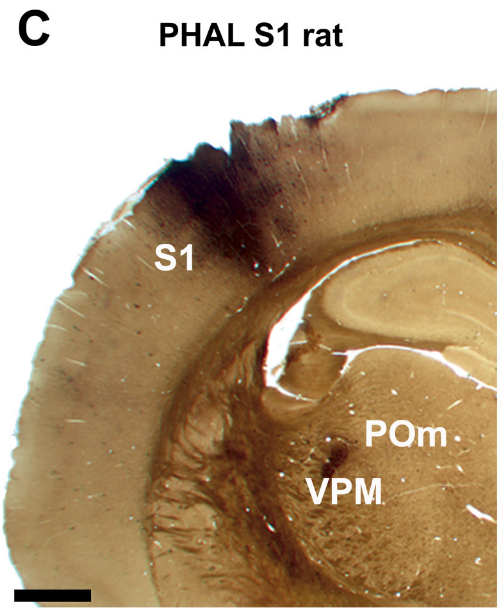

B Table 1

No. of section	No. of layer 5 boutons in POm: in vGlut2 rich area (Σ:178)	No. of layer 5 boutons in POm: in vGlut2 poor area (Σ:236)	% of vGlut2 rich area in POm
1	136	0	46.6
2	16	0	39.1
3	0	96	18.5
4	0	81	19.7
5	50	53	16.9
6	21	6	13.7

Table 2

animal	Proportion of vGlut2 rich zones in POm (%)	Proportion of large cortical terminals located in vGlut2 rich zones in POm (%)	n (No. of large cortical terminals examined)
1	32.34	22.12	321
2	22.84	68.84	292
3	27.37	43	414

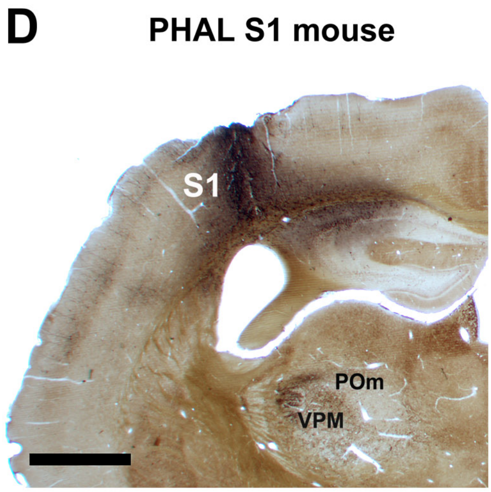

Driver terminals in POm

A) Method of mapping the distribution of giant cortical and subcortical excitatory (driver) terminals in POm of rats. The distribution of large cortical (black dots, 1 dot = 2 boutons) and vGlut2-positive subcortical (yellow outline) terminals was examined in representative sections of POm (blue outline). Yellow outline indicate vGlut2 rich zones. vGLUT2 rich zones were operationally defined as having more than 15 vGLUT2-positive on the top and bottom surface of the section together, measured within a 100x100 µm area (green rectangles), examined by a x63 oil immersion objective with 1.4 numerical aperture. The numbers withing the green rectangles indicate the actual number of vGLUT2-positive terminals.

B) Table 1 and 2 show the quantification of the distribution of vGlut2-positive and the S1 layer 5 terminals in the POm of a representative animal (rat, same as in A and Figure 1).

C) Injection site of Phaseolus vulgaris leucoagglutinin (PHAL) in the primary somatosensory cortex (S1) of rat, and D) mouse.

Alternative Pathways

S1 ChR2 activation may induce polysynaptic activation and can indirectly lead to response increase in POm. Her we review the output pathways of S1 which may be involved and argue that they can not account for the fast monosynaptic effect we observe in POm.

Striatum, basal ganglia

We are currently not aware of any major input from the basal ganglia to POm or basal ganglia output to the input region of POm. Furthermore, basal ganglia itself is polysynaptic hence we don't think alternative pathways via the basal ganglia is possible.

S1-M1

Based on retrograde tracing the available literature suggests (Veinante et al., 2000 JCN 424:197) that POm receives L5B input from S1 and not from M1. Confirming this, following anterograde tracing from M1 we found only small terminals in POm, never large ones (we can provide the data for reviewing purposes). The lack of large terminals from M1 makes the measured large effect unlikely. But even if drivers of POm from M1 exists and can be recruited polisynapticaly our core discovery would be still valid (i.e convergence of drivers with different origin on the same thalamocortical cell.)

S1-M1-Zi

Urbain and Deschenes (Neuron 56:714) suggested an interesting scenario, how transmission of trigeminal input can be facilitated in POm, which is otherwise strongly attenuated via an inhibitory pathway from ZI. They claim that M1 input can activate the motor sector of ZI, which would in turn inhibit the somatosensory sector of ZI, hence disinhibitng POm cells. Since S1 projects to M1, In principle, sensory transmission can be facilitated in POm via this polsynaptic pathway. However,

a) This network contains five synapses from S1 to POm (S1, M1, motor ZI, somatosensory ZI, POm). Reliable transmission is not realistic through five synapses especially within 10 ms.

b) We found EPSPs (and occasionally IPSPs) after S1 stimulation (Figure 8) not the lack of IPSPs which would be suggested by a disinhibitory pathway.

c) There is a strong and direct S1-somatosensory ZI pathway (Barthó et al., 2007 JNsci 27:1670), which would actually work against the direct excitatory S1-POm pathway, since it activates the inhibitory ZI-POm pathway (Bartho et al., 2002). The S1-ZI effect is probably much stronger than the indirect M1-ZI pathway. In fact, intracellular recordings, mentioned above occasionally reveal IPSPs in POm after S1 stimulation probably via this pathway. Nevertheless S1, trigeminal convergence evokes a robust effect. As we discuss one reason of the convergence may be to overcome the ZI inhibition.

Whisker-VPM-S1

This pathway is considered in the discussion (line 524-549). Briefly whisker input does not have enough time to reach S1 L5B within 10 ms to be able to alter laser evoked L5B output.
